# Cerebellar Paired Associative Stimulation Enhances Motor Learning and Modulates Cerebellar Output in a Timing- and Task-Dependent Manner

**DOI:** 10.1007/s12311-026-01957-9

**Published:** 2026-02-12

**Authors:** Damiano Sottana, Danny A. Spampinato, Mohammed Zeroual, Nicola Loi, Matteo Spinelli, Francesca Ginatempo, Franca Deriu

**Affiliations:** 1 Department of Biomedical Sciences, University of Sassari, Viale San Pietro 43/b, Sassari, 07100 Italy; 2 Department of Clinical and Behavioral Neurology, IRCCS Santa Lucia Foundation, Rome, Italy; 3 Saint Camillus International University of Health and Medical Sciences (UniCamillus), Rome, Italy; 4 Azienda Ospedaliero Universitaria di Sassari, AOU Sassari, Sassari, Italy; 5 Unit of Endocrinology, Nutritional and Metabolic Disorders, AOU Sassari, Sassari, Italy

**Keywords:** Cerebellum plasticity, Motor learning, Transcranial magnetic stimulation, Paired associative stimulation, Neuromodulation, Sensorimotor integration

## Abstract

Cerebellar paired associative stimulation (cPAS) is a non-invasive neuromodulation protocol that combines peripheral nerve stimulation with cerebellar transcranial magnetic stimulation to induce plasticity in the cerebellar-cortical pathway. Previous evidence suggested that cPAS delivered at a 25ms interstimulus interval (cPAS25) can modulate cerebellar-primary motor cortex connectivity, as reflected by changes in cerebellar–brain inhibition (CBI). However, its behavioural relevance remains unclear. To investigate this issue, we conducted two experiments in young healthy adults using a within-subject crossover design to compare the effects of cPAS25 and a temporally mismatched control (cPAS10). In Experiment 1, participants received cPAS followed by a visuomotor sequence learning task, with motor performance assessed via movement time, error rate, and a composite skill index. CBI and motor evoked potentials (MEPs) were recorded at baseline, post-stimulation, and after task completion. In Experiment 2, a subset of participants received cPAS without the task to isolate its neurophysiological effects. cPAS25 significantly improved motor learning compared to cPAS10, as shown by a greater increase in skill index. It also reduced CBI, but this effect was observed only when cPAS was not followed by motor practice, suggesting a task-sensitive interaction. MEP amplitudes remained unchanged, indicating selective modulation of cerebellar output. These results support a timing-dependent, context-sensitive mechanism of cerebellar plasticity. cPAS25 can enhance motor learning and modulate cerebellar–cortical connectivity, although effects may not summate when paired with motor practice. These findings highlight the translational potential of cPAS25 as a precision neuromodulatory approach to enhance motor learning and rehabilitation by targeting cerebellar circuits.

## Introduction

Motor learning relies on the dynamic interaction between the cerebellum and the primary motor cortex (M1). The cerebellum is essential for sensorimotor integration, error correction, and the predictive control of movement, mainly through projections to M1 via the dentate–thalamo-cortical pathway [1, 2]. Through these circuits, the cerebellum continuously modulates motor output in response to incoming sensory feedback. This dynamic loop supports both movement execution and motor learning, underpinned by plastic changes in cortical and subcortical circuits. Structural and functional adaptations in M1 during motor skill acquisition have been demonstrated using both neuroimaging and non-invasive stimulation methods [3–5].

Beyond its role in motor execution, the cerebellum is increasingly recognized as a modulator of plasticity in M1. [6] demonstrated that cerebellar excitability can bidirectionally influence M1 plasticity: cerebellar inhibition enhanced and prolonged paired associative stimulation (PAS)-induced Long-Term Potentiation (LTP)-like effects in M1, whereas cerebellar excitation attenuated them. These findings suggest that cerebellar processing of afferent input can gate cortical plasticity, likely via dentate and/or thalamic relay structures.

PAS protocols are essential tools for studying spike-timing dependent plasticity in humans. Classical PAS involves stimulating a peripheral nerve followed by TMS over M1 at specific interstimulus intervals (ISIs), resulting in LTP- or Long-Term Depression (LTD)-like changes in corticospinal excitability, depending on the ISI used [7, 8]. This method has been adapted to cerebellar circuits through cerebellar PAS (cPAS), where peripheral input is paired with TMS over the cerebellum [9]. When delivered at a 25 ms ISI (cPAS25), this protocol decreases cerebellar–brain inhibition (CBI), a physiological marker of cerebellar–cortical connectivity [9].

CBI, assessed with dual-site TMS protocol, reflects inhibitory Purkinje cell output to M1 via the deep cerebellar nuclei and thalamus [10]. Notably, changes in CBI have been associated with motor adaptation and learning [11] 2018). Other cerebellar stimulation methods, such as theta burst stimulation, have also been shown to modulate both CBI and motor performance, reinforcing the cerebellum’s role in adaptive motor control [12].

Temporal specificity appears to be critical for cPAS efficacy. Effective modulation of CBI occurs only at ISIs between 25 and 35 ms [13], suggesting a narrow time window for cerebellar–cortical plasticity. Consistent with this finding, our recent work showed that CBI was significantly reduced by cPAS25, but not by a temporally mismatched protocol using a 10 ms ISI (cPAS10) [9].

However, whether such physiological changes translate into improved motor learning remains unclear. Establishing this link could guide the design of cerebellar-targeted neuromodulation protocols for enhancing motor recovery in clinical populations. Therefore, in this study we investigated the behavioural and neurophysiological consequences of cPAS25 versus cPAS10 in young healthy adults, using a within-subject crossover design, where each cPAS protocol was followed by a visuomotor sequence learning task. We hypothesized that cPAS25 would enhance cerebellar–M1 plasticity, improving motor learning and reducing CBI. Demonstrating this link would provide a foundation for cerebellar-targeted neuromodulation strategies to support motor rehabilitation.

## Methods

### Participants

Twenty-three young healthy subjects, all right-handed according to the Oldfield inventory scale, were recruited for the study (10 females, mean age 26.4 ± 5.3 years). The sample size was based on an a priori analysis using repeated-measure within subjects factors ANOVA with G*Power 3.1 software assuming an expected effect size d = 0.6, two independent group and a statistical power of 0.80 at a 0.05 alpha level which resulted in a total sample size of 23 subjects. All participants were tested for TMS exclusion criteria [14]. None had a history or current signs or symptoms of neurological or psychiatric diseases, sleep disorders, or ongoing or past pharmacological therapy. The experimental procedure was approved by the local ethical committee (PROT. PG/2021/5435) and conducted in accordance with the Helsinki Declaration. Informed consent was obtained from all participants.

### Electromyography (EMG)

Surface EMG was recorded from the left first dorsal interosseous (FDI) muscle using 9 mm diameter Ag-AgCl surface electrodes. The active electrode was settled on the muscle belly, the reference electrode at the second finger metacarpophalangeal joint, and the ground electrode over the forearm bony prominence [15]. EMG signals were recorded (D360 amplifier, Digitimer Ltd), amplified (×1000), filtered (bandpass 3–3000 Hz), and sampled at 5 kHz using a 1401 power analog-to-digital converter and Signal 6 software (Cambridge Electronic Design).

## Transcranial Magnetic Stimulation (TMS)

Cortical stimulation was delivered using a figure-of-eight coil (7 cm external loop diameter) attached to a Magstim 200 stimulator (Magstim Co., Whitland, Dyfed, UK). The precise stimulation site for the non-dominant, left FDI muscle was carefully identified and marked to ensure consistent coil positioning. The coil handle was oriented posteriorly and laterally at approximately a 45° angle to the interhemispheric line [16]. Resting motor threshold (RMT) was defined as the lowest TMS intensity that elicited motor evoked potentials (MEPs) of ≥ 0.05 mV in at least 5 out of 10 consecutive trials. CBI was assessed at rest condition and not during the behavioral task using a paired-pulse, dual-coil TMS protocol. The conditioning stimulus (CS) was delivered via a double cone coil (7 cm external loop diameter) connected to a second Magstim 200 stimulator. The coil was placed over the cerebellum, 3 cm lateral to the inion and contralateral to the M1 stimulation site. CS intensity was set at 60% of the maximum stimulator output (MSO) in accordance with a previous study (Ginatempo et al., 2024). The test stimulus (TS) intensity was adjusted to evoke MEPs of approximately 1 mV (mvMEP), with an ISI between CS and TS of 5ms. Fifteen unconditioned and fifteen conditioned MEPs were recorded in randomized order. CBI was calculated as the ratio between conditioned and unconditioned MEP amplitudes.

## Cerebellar Paired Associative Stimulation Protocols (cPAS)

The cPAS protocol consisted of 200 paired stimuli. An electrical stimulus was delivered over the median nerve of the left arm at an intensity of three times the subjective perceptual threshold, followed by a TMS pulse delivered over the ipsilateral cerebellar hemisphere with a double cone coil, and intensity set at 60% of the MSO. Two different ISIs were used: 25ms as the “real” protocol, and 10ms as the “control”.

## Sequential-Visual-Isometric-Pinch Task (SVIPT)

Motor learning was assessed with the SVIPT. The SVIPT task is a well-established paradigm for probing the distinct temporal components of motor-skill learning [17] and is therefore well suited for investigating cerebellar involvement. Notably, the SVIPT requires learning a nonlinear force-to-cursor transformation, precise temporal scaling of force, and continuous sensorimotor prediction, which are features known to strongly engage cerebellar computations [18–20].

The task was performed with the non-dominant left hand, to avoid a possible ceiling effect. Participants used a precision pinch to move a cursor on a screen to sequentially reach five numeric targets. Force was measured via a P200 transducer (P200 Biometrics Ltd, Newport, UK) to move a cursor (a black square) to reach five different targets organized in a specific numeric sequence. Participants held an isometric force transducer between the right thumb and index finger to control an on-screen cursor. Cursor movement was logarithmically scaled to pinch force to increase task difficulty and extend learning. Force-to-cursor mapping parameters, including the maximum force for rightward movement, matched those used by [17]. Subjects moved the cursor between a HOME position and five sequential gates (HOME-1-HOME-2-HOME-3-HOME-4-HOME-5) by modulating pinch force. A STOP signal appeared once Gate 5 was reached and held, marking trial completion. Participants were instructed to perform as quickly and accurately as possible. Apart from repeated task instructions, no additional verbal feedback was given. The task consisted of three 30-trial blocks: one familiarization block and two test blocks (blocks 2 and 3) used for statistical analysis. Participants were seated with elbows at 90°, with forearms supported and in a neutral position and instructed to minimize errors and movement time. Performance was quantified using error rate, movement time, and skill index (see formula in Statistical Analysis’ section). The task followed previously described protocols [17, 21].

## Experimental Design

**Fig. 1:**
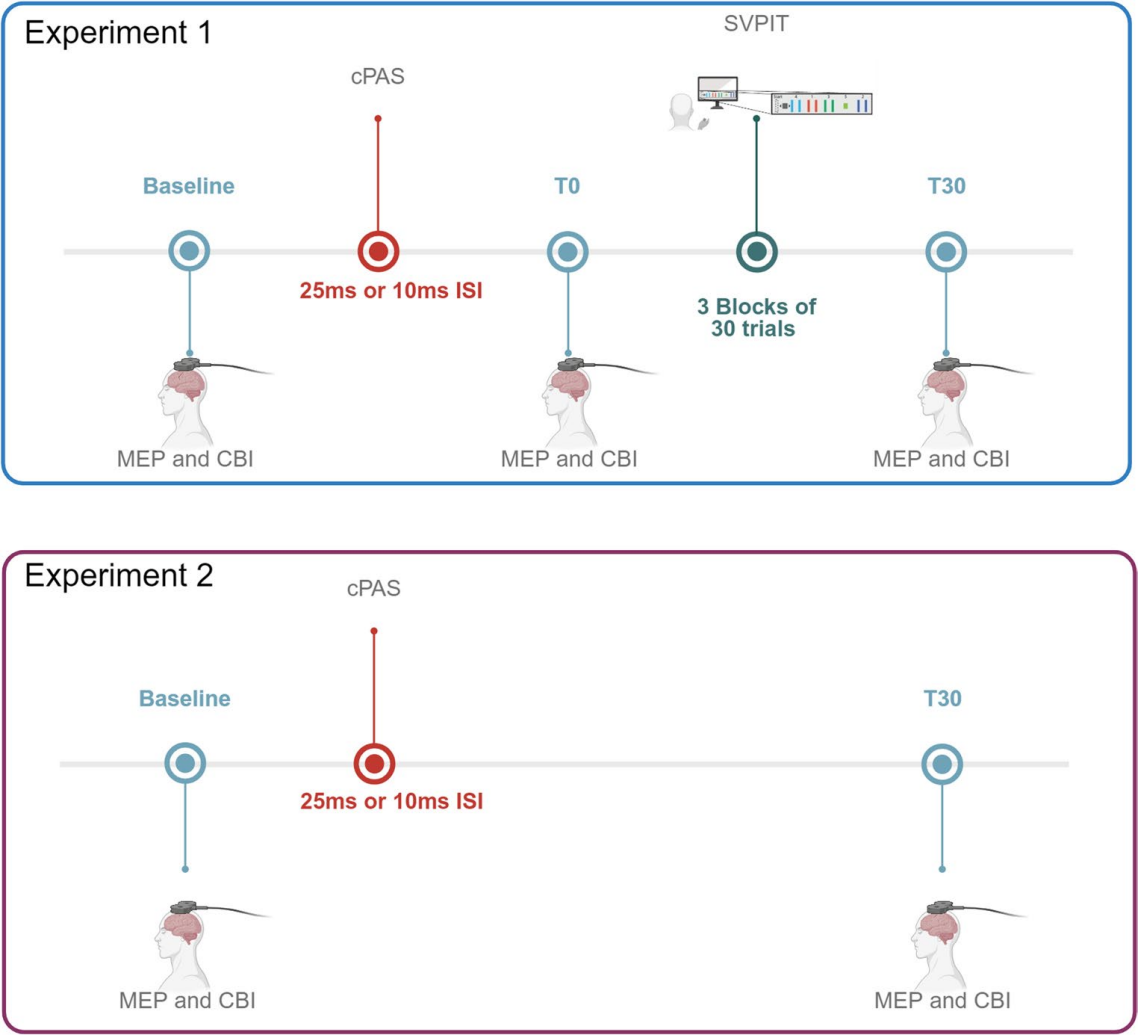
Experimental design. The experimental design included two experiments, which were held at least one week apart. In experiment 1 participants underwent a baseline evaluation of motor evoked potentials (MEP), and cerebellar brain inhibition (CBI) recorded from the first dorsal interosseus (FDI) muscle of the left hand. After the baseline neurophysiological assessment, cerebellar paired-associative stimulation (cPAS) was delivered with interstimulus intervals of 25ms as the “real” protocol and 10ms as a control, in two sessions separated by at least one week. Subsequently, participants underwent assessment of MEPs and CBI at T0 (i.e., immediately after cPAS), assessment of motor learning through the sequential visuo-isometric pinch task (SVIPT) and assessment of MEPs and CBI at T30 (i.e., after 30 min from cPAS delivery). Finally, MEPs and CBI were assessed after the SVIPT at T30 (after 30 min from cPAS delivery). Experiment 2 consisted of the assessment of MEPs and CBI at baseline and 30 min after cPAS delivery. This experiment was composed of two separate sessions for the delivery of cPAS25 and cPAS10, between which at least one week elapsed. Created in https://BioRender.com

The effect of different ISIs of cPAS was evaluated on motor performance and CBI in two experimental sessions performed one week apart. Motor performance was assessed via movement time, error rate, and a composite skill score. CBI was measured before and after stimulation and after task completion. An additional control experiment was performed only with neurophysiological evaluations in a subset of subjects, with at least one week separating it from the main experiment (Fig. 1).

### Experiment 1. Behavioral and neurophysiological assessments following the administration of cPAS at 25ms ISI (cPAS25) and at 10ms ISI (cPAS10

All subjects participated in two experimental sessions, one for each ISI. MEP and CBI were measured at three different time points: before cPAS delivery (baseline), immediately after cPAS delivery (T0), and after motor learning (30 min after cPAS, T30). Between T0 and T30, participants completed the SVIPT. The administration of cPAS25 or cPAS10 was counterbalanced for each subject.

### Experiment 2. Neurophysiological assessment following the administration of cPAS at 25ms ISI (cPAS25) and at 10ms ISI (cPAS10)

To eliminate any potential confounding effects of SVIPT on the neurophysiological parameters and to replicate the effect of CBI observed in a previous study (Ginatempo et al., 2024), eleven subjects (5 females, with a mean age of 30.9 ± 4.9 years) out of the twenty-three who participated in the first experiment took part in experiment 2, which was designed to isolate the neurophysiological effects that, according to a previous study, was observed only after 30 min from cPAS delivery [9]. This consisted of two experimental sessions, one for each ISI, during which MEP and CBI were measured before (baseline) and 30 min (T30) after cPAS (cPAS25 or cPAS10) delivery. The administration of cPAS25 or cPAS10 was counterbalanced for each subject.

#### Data and Statistical Analysis

*Data analysis.* Data analysis was performed offline with Signal 6.06 software (Cambridge Electronic Design Ltd, UK). Peak-to-peak MEP amplitude and the ratio of conditioned to unconditioned MEP amplitude were calculated and used as a variable in MEP and CBI protocols. To assess motor learning, the mean error rate and movement time for each trial and block of the SVIPT were analyzed as variables. Additionally, a skill index for each block was used to calculate the learning effect using the following formula [17, 22]:

$$\:Skill=\frac{1-\frac{Errors}{30}}{Movement\:time}$$

Since the first trial was used as a familiarization trial, an overall skill index was calculated by normalizing for the first block using the following formula:

$$\:Total\:Skill=\frac{Skill\:Block\:3-Skill\:Block\:2}{Skill\:Block\:1}$$

*Statistical analysis*. Statistical analyses were performed with SPSS 20.0 (SPSS Inc., Chicago, IL, USA). Normality was assessed by the Kolmogorov-Smirnov test. Sphericity was tested with Mauchly’s test; if sphericity was violated (i.e., Mauchly’s test < 0.05), the Greenhouse-Geisser correction was applied. Comparisons were made using Student’s paired t-test, repeated-measures of analysis of variance (RM-ANOVA), and planned post-hoc t-tests with Bonferroni correction. Statistical significance was defined as *p* < 0.05. Data are reported as mean ± standard error of the mean (SEM).

Experiment 1: To determine whether significant inhibition occurred at baseline in the CBI protocol and whether differences existed between cPAS conditions, a preliminary RM-ANOVA was conducted using raw MEP amplitudes at baseline as the dependent variable.

To assess the reliability of CBI measurements across cPAS sessions, we computed the intraclass correlation coefficient (ICC). In addition, to evaluate whether baseline CBI differed across sessions, we performed a RM-ANOVA on the baseline CBI ratio for each cPAS session (cPAS10 from the first experiment, cPAS25 from the first experiment, cPAS10 from the second experiment, and cPAS25 from the second experiment), with session as the within-subject factor.

Subsequently, a two-way RM-ANOVA was performed with cPAS condition (cPAS25, cPAS10) and ISI (test MEP; conditioned MEP for CBI) as within-subjects factors. If a significant main effect of ISI was detected, separate two-way RM-ANOVAs were conducted on both raw MEP amplitudes and CBI ratios, using cPAS (cPAS25, cPAS10) and TIME (baseline, T0, T30) as within-subjects factors.

A preliminary paired t-test was also conducted to compare error rate, movement time, and skill index in the familiarization block of SVIPT between the cPAS10 and cPAS25 sessions. If no significant difference was found, two additional two-way RM-ANOVAs were conducted on error rate, movement time and skill index with cPAS (cPAS25, cPAS10) and block (Block 2, Block 3) as within-subjects factors.

Finally, a paired t-test was used to compare the total skill index between cPAS25 and cPAS10 sessions.

Experiment 2: To assess whether significant inhibition was present at baseline in the CBI protocol, and to examine potential differences between cPAS sessions, a preliminary RM-ANOVA was conducted using baseline raw MEP amplitude as the dependent variable.

Following this, a two-way RM-ANOVA was conducted with cPAS condition (cPAS25, cPAS10) and ISI (test MEP; conditioned MEP for CBI) as within-subjects factors. If a significant main effect of ISI was observed, two additional two-way RM-ANOVAs were performed separately on raw MEP amplitudes and CBI ratios, using cPAS (cPAS25, cPAS10) and TIME (baseline, T30) as within-subjects factors.

## Results

The statistical analysis failed to detect any significant effect at baseline between the two sessions on raw amplitude MEP and CBI ratio (Table 1).

**Table 1:** Neurophysiological parameters at baseline in the cPAS25 and cPAS10 sessions

Neurophysiological variables	*Experiment 1 (n = 23)*		Statistics
	cPAS25	cPAS10	
Raw MEP amplitude	1.18 ± 0.25	1.09 ± 0.19	*p* = 0.920
CBI ratio	0.77 ± 0.06	0.81 ± 0.07	*p* = 0.525
	*Experiment 2 (>= 11)*		
Raw MEP amplitude	1.65 ± 0.29	1.77 ± 0.35	*p* = 0.82
CBI ratio	0.68 ± 0.07	0.79 ± 0.08	*p* = 0.24

*cPAS* cerebellar paired associative stimulation, in *cPAS* 25 and 10 indicate the interstimulus time interval in ms, *RMT* resting motor threshold, *MEP* motor evoked potentials, *CBI* cerebellar-brain inhibition. Data are represented as mean ± SEM. *p < 0.05

### Experiment 1 Behavioral and neurophysiological assessment following the administration of cPAS at 25ms ISI (cPAS25) and at 10ms ISI (cPAS10)

ICC analysis detected a moderate reliability of CBI measurement among cPAS25 and cPAS10 sessions (ICC = 0.532; *p* = 0.038). RM-ANOVA on CBI ratio differences across sessions, showed no significant session effect (F_3,30_ = 0.334, *p* = 0.701).

Statistical analysis did not reveal any significant effects of cPAS on M1 excitability or cerebellar–M1 connectivity. However, cPAS25 significantly improved skill index although it did not affect error rate and movement time.

**Fig. 2:**
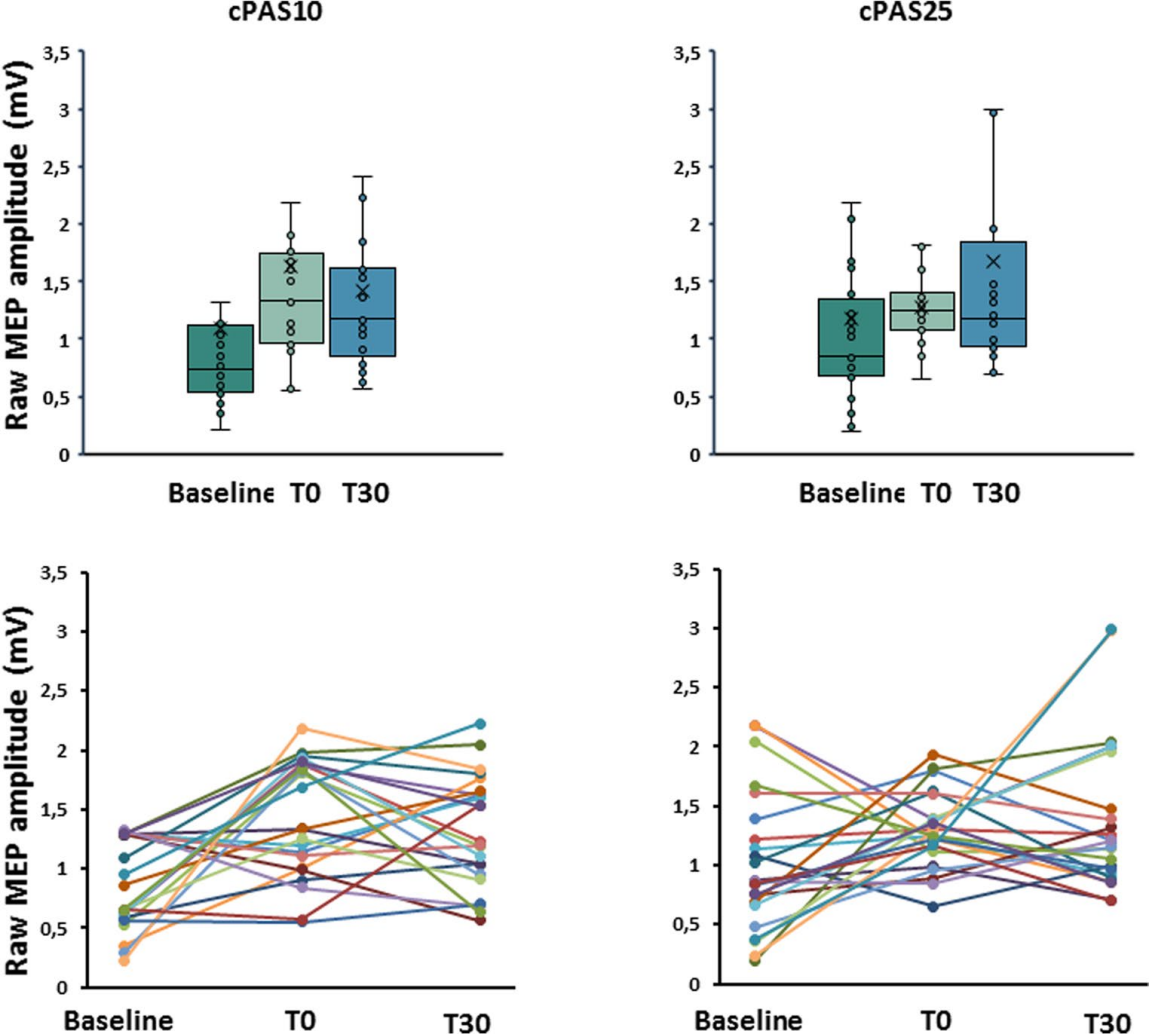
Effects of the cPAS and motor learning protocols on motor cortex excitability. Graphs showed the raw motor evoked potentials (MEP) amplitude at baseline and immediately after (T0) and after 30 min (T30) from cPAS delivery at interstimulus intervals of 10 and 25ms (cPAS10 and cPAS25, respectively). The upper panels display group variability using box plots, while the lower panels show individual trends across the analyzed time points. The continuous line in the boxplot represents the median value, while the ‘×’ symbol represents the mean value of the group. Dots represent individual data. *p < 0.05

*MEP.* RM-ANOVA showed no significant effects of TIME (F_1,22_=2,689; *p* = 0.092; η^2^*p*=0.113), cPAS (F_1,22_=0.024; *p* = 0.879; η^2^*p*=0.001), and interaction between factors (F_1,22_=1.683; *p* = 0.199; η^2^*p*=0.074) (Fig. 2).

**Fig. 3:**
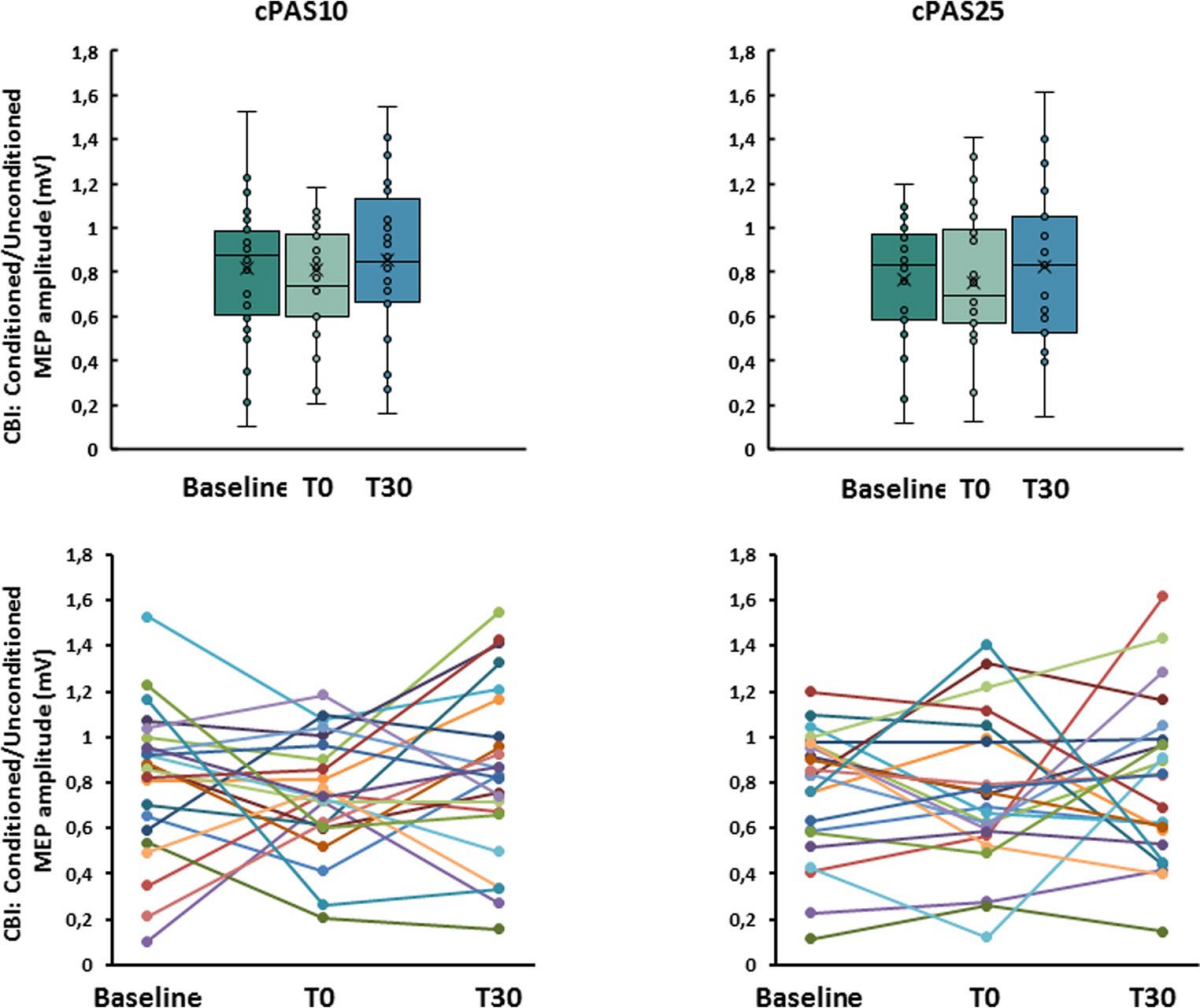
Effects of the cPAS and motor learning protocols on cerebellar-brain inhibition (CBI). Graphs showed the ratio between the amplitudes of conditioned and unconditioned motor evoked potentials (MEP) during CBI paired pulse protocol at baseline, immediately after (T0) and after 30 min (T30) from cPAS delivery at interstimulus interval 10 and 25 ms (cPAS10 and cPAS25, respectively). The upper panels display group variability using box plots, while the lower panels show individual trends across the analyzed time points. The continuous line in the boxplot represents the median value, while the ‘×’ symbol represents the mean value of the group. Dots represent individual data. *p < 0.05

*CBI.* The preliminary analysis demonstrated the presence of cerebellar inhibition at baseline, with no significant difference between sessions. A two-way RM-ANOVA showed a significant effect of ISI (F_1,22_=17.697; *p* < 0.001; η^2^*p*=0.446) but non-significant effects of cPAS (F_1,22_=0.002; *p* = 0.968; η^2^p = < 0.001) or interaction between factors (F_1,22_=0.348; *p* = 0.561; η^2^*p*=0.016). Subsequent analysis on the CBI ratio using RM-ANOVA showed no effects of TIME (F_1,22_=0.299; *p* = 0.737; η^2^*p*=0.015), cPAS (F_1,22_=1.203; *p* = 0.286; η^2^*p*=0.057) or their interaction (F_1,22_=0.104; *p* = 0.896; η^2^*p*=0.005) (Fig. 3).

*SVIPT.* In the familiarization block (Block 1), no significant differences were found between cPAS10 and cPAS25 for movement time (t_1,22_*=*1.451; *p* = 0.161), error rate (t_1,22_*=*0.480; *p* = 0.636) or skill index (t_1,22_*=*−0.325; *p* = 0.748).

**Fig. 4:**
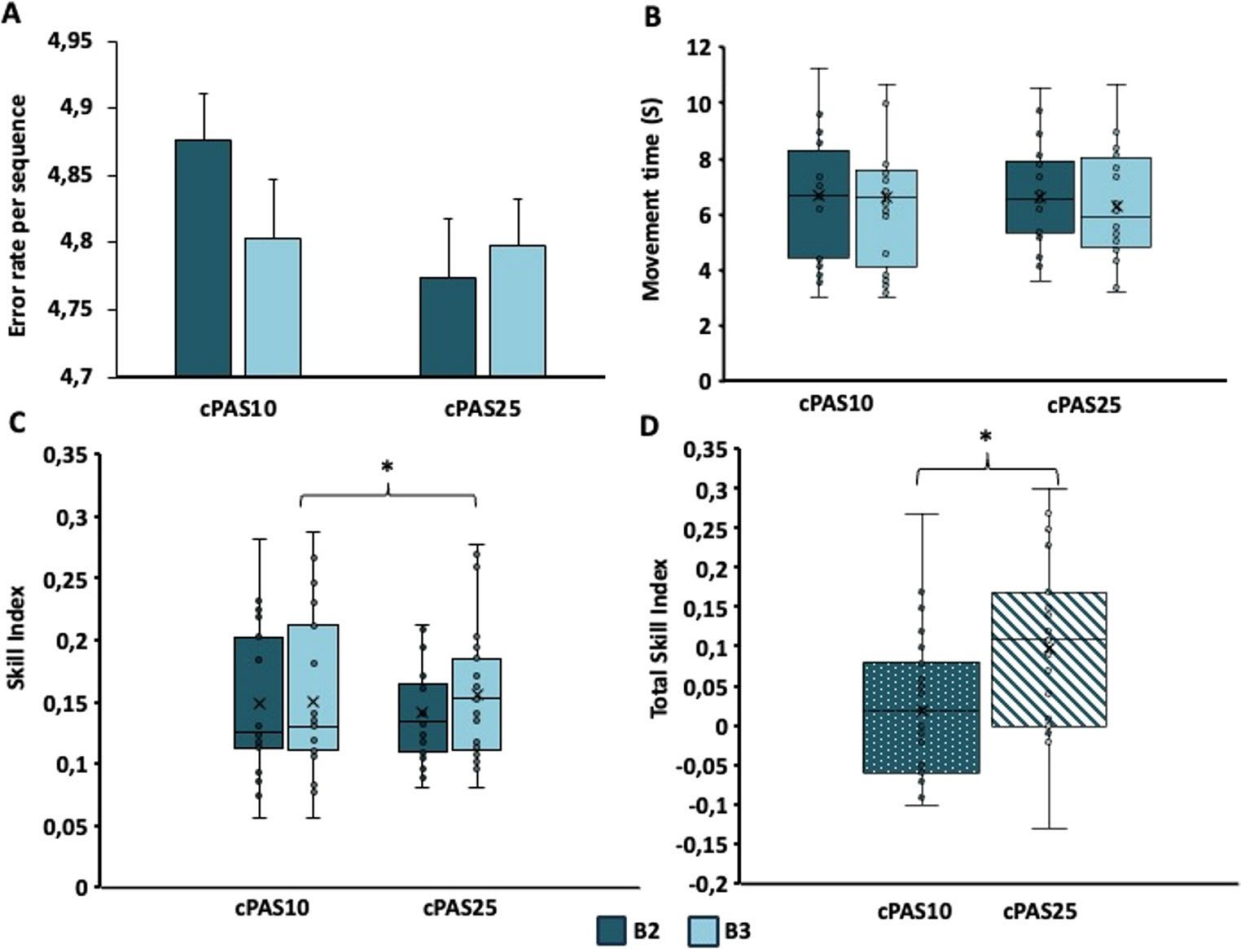
**Effect of the cPAS on error rate**,** movement and skill index during motor learning.** In Panel A, histograms show the error rate for the second (B2) and the third (B3) block of trials after cPAS delivery at both interstimulus intervals of 10 and 25ms (cPAS10 and cPAS25, respectively). cPAS10 showed a significant learning effect that was not observed following cPAS25. The histograms report means ± standard error of the mean; *p < 0.05. The box plots, show the movement time (panel B), the skill index (panel C) and the total skill index (panel D) for B2 and B3 after cPAS delivery at interstimulus intervals of 10 and 25ms (cPAS10 and cPAS25, respectively). In the box plots, the continuous line represents the median value, while the ‘×’ symbol represents the mean value of the group. Dots represent individual data. *p < 0.05

*Error Rate.* RM-ANOVA revealed a significant effect of block (F_1,22_=5.587; *p* = 0.027; η^2^*p*=0.203) but no effect of cPAS (F_1,22_=2.232; *p* = 0.149; η^2^*p*=0.092) and no interaction (F_1,22_=0.427; *p* = 0.520; η^2^*p*=0.019). Post-hoc analysis showed a significant reduction of error rate between Block 3 and Block 2 (*p* = 0.027) (Fig. 4A).

*Movement Time.* A significant main effect of block was found (F_1,22_=6.656; *p* = 0.017; η^2^*p*=0.232), with no significant effect of cPAS (F_1,22_=1.031; *p* = 0.321; η^2^*p*=0.045) and a trend toward interaction (F_1,22_=3.938 *p* = 0.060; η^2^*p*=0.152). Post hoc analysis confirmed movement time reduction in Block 3 vs. Block 2 (*p* = 0.017) (Fig. 4B).

*Skill Index.* RM-ANOVA revealed no effect of cPAS (F_1,22_=0.004; *p* = 0.953; η^2^p = < 0.001), but a significant main effect of block (F_1,22_=14.295; *p* = 0.001; η^2^*p*=0.394) and interaction between factors (F_1,22_=4.912; *p* = 0.037; η^2^*p*=0.183). Post-hoc analysis of the main factor showed an improvement of the skill index in Block 3 compared to Block 2 (*p* = 0.001). Moreover, post-hoc analysis of the interaction showed a significant improvement only following cPAS25 (*p* < 0.001) (Fig. 4C).

*Total Skill Index.* A paired t-test showed a significant effect of cPAS on total skill index, which was significantly higher only following cPAS25 (t_1,22_=−2.296; *p* = 0.032) (Fig. 4D).

### Experiment 2. Neurophysiological assessment following the administration of cPAS at 25ms ISI (cPAS25) and at 10ms ISI (cPAS10)

CBI was significantly reduced at 30 min post-stimulation only following cPAS25.

*MEP.* RM-ANOVA showed no effects of TIME (F_1,10_=0.058; *p* = 0.815; η^2^*p*=0.006), cPAS (F_1,10_<0.001; *p* = 0.999; η^2^p = < 0.001) or interaction between factors (F_1,10_=0.212; *p* = 0.655; η^2^*p*=0.021).

**Fig. 5:**
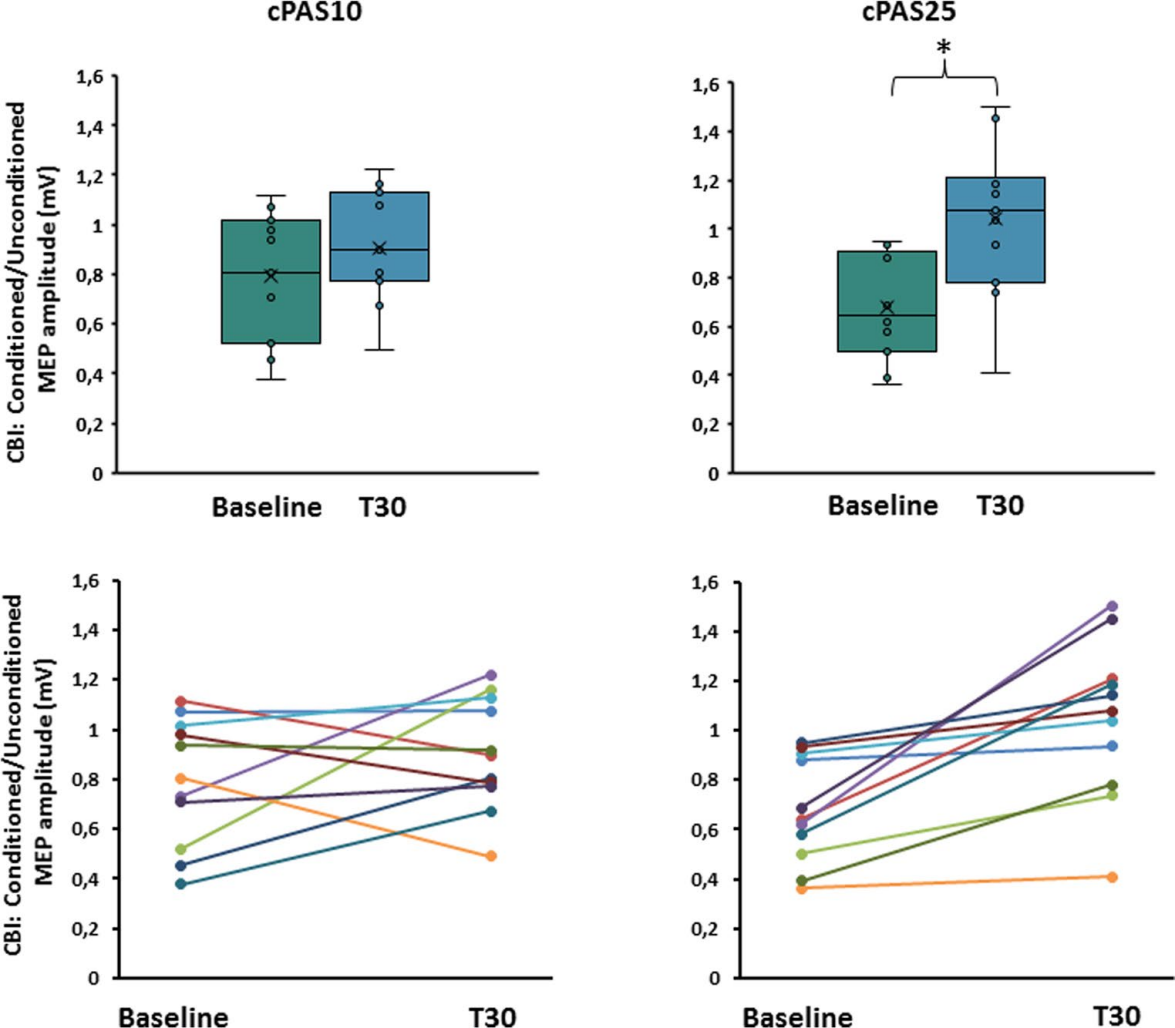
Effects of the cPAS and motor learning protocols on cerebellar-brain inhibition (CBI). Graphs show the ratio between conditioned and unconditioned motor evoked potentials (MEP) amplitude during CBI paired pulse protocol before (baseline) and after 30 min (T30) from cPAS delivery at interstimulus intervals of 10 and 25ms (cPAS10 and cPAS25, respectively). The upper panels display group variability using box plots, while the lower panels show individual trends across the analyzed time points. The continuous line in the boxplot represents the median value, while the ‘×’ symbol represents the mean value of the group. Dots represent individual data. *p < 0.05

*CBI.* As in Experiment 1, significant inhibition of MEP amplitude occurred at baseline, with no session differences. Two-way RM-ANOVA revealed a significant effect of ISI (F_1,10_=17.482; *p* = 0.002; η^2^*p*=0.636) but a no significant main effect of cPAS (F_1,10_=0.192; *p* = 0.671; η^2^*p*=0.019) or interaction between factors (F_1,10_=0.993; *p* = 0.343; η^2^*p*=0.090). CBI ratio analysis showed no significant effect of cPAS (F_1,10_=0.027; *p* = 0.873; η^2^*p*=0.003) but a significant effect of TIME (F_1,10_=10.588; *p* = 0.009; η^2^*p*=0.514) and interaction between factors (F_1,10_=5.735; *p* = 0.038; η^2^*p*=0.364). Bonferroni-corrected post-hoc testing confirmed a significant reduction of CBI only following cPAS25 (*p* = 0.002) (Fig. 5).

## Discussion

Our findings demonstrate that cerebellar paired associative stimulation at a 25ms ISI (cPAS25) can facilitate motor learning in young healthy adults, as shown by improved performance on a SVIPT task following stimulation. Moreover, a significant reduction in CBI suggests that cPAS25 can modulate cerebellar–cortical connectivity in a timing-specific manner. This effect was observed when compared with a temporally mismatched protocol (cPAS10), which failed to produce changes in motor performance or CBI, underscoring the spike-timing-dependent nature of the induced plasticity.

The specificity of the effect to the 25ms ISI is particularly compelling. Previous studies have shown that interstimulus intervals between 25–35ms are optimal for modulating cerebellar–cortical connectivity, likely reflecting natural conduction delays in sensorimotor pathways [13]. Our results confirm this hypothesis, demonstrating both a behavioural benefit and CBI modulation following cPAS25, but not cPAS10. These findings underscore the critical role of temporal precision in cerebellar PAS protocols and align with broader evidence of spike-timing dependent plasticity in human sensorimotor systems [7, 8].

To our knowledge, this is the first study demonstrating that cerebellar PAS can enhance behavioural performance on the SVIPT task, supporting the cerebellum’s role as a modifiable node in motor learning circuits. Although an improvement was observed in the skill index, this effect was not reflected in movement time or error rate considered individually. The skill index takes both variables into a single measure based on the speed-accuracy trade-off and is therefore more reliable for capturing motor learning-related changes. This approach avoids confounding patterns in which reduced movement time is accompanied by increased errors (or vice versa). Thus, even if small changes in movement time or error rate occurred independently (though not statistically significant), their combined contribution produced a detectable improvement in the skill index. This interpretation aligns with previous studies using this task [17, 23]. Importantly, the SVIPT task used here engages fine sensorimotor control and error-based adaptation, processes known to involve both M1 and the cerebellum [21, 23–25]. Prior studies have shown that motor learning induces structural and functional changes in both M1 and cerebellum [5, 26, 27] and learning-related modulation of cortical excitability varies by training phase [4]. Our findings extend this work by demonstrating that cerebellar-targeted PAS can prime these circuits to enhance subsequent learning, via modulation of CBI.

Mechanistically, we propose that cPAS25 induces plasticity at the parallel fiber–Purkinje cell synapse within the cerebellar cortex. This synapse integrates contextual input from mossy fibers carried via the spino-cuneo-cerebellar tract and error signals from climbing fibers, which arise through the spino-inferior olivary (IO) pathway [28–30]. The 25ms ISI likely reflects the physiological conduction delay from peripheral stimulation to cerebellar processing, making cPAS25 well-suited to engage LTD-like mechanisms at Purkinje cells. This would decrease Purkinje cell output and thereby decrease CBI, consistent with our findings. Previous work has demonstrated that cerebellar stimulation can modulate both behavior and physiology [12, 31–33], and that cerebellar excitability can shape cortical plasticity [6]. Importantly, [34] further showed that cerebellar activity modulates associative plasticity in M1, supporting our interpretation that cerebellar PAS can engage downstream cortical mechanisms.

A key insight from our study is that the reduction in CBI was observed in the control condition where cPAS25 was delivered alone, but not when it was followed by motor learning. One possible explanation is that the cerebellar–cortical pathways are activated by both stimulation and the task, saturating plasticity [35, 36], which suggests limited plastic potential in each circuit [6, 11]. Alternatively, task-related plasticity may override or mask the effects of cPAS, particularly if both rely on similar mechanisms such as error-based learning and Purkinje cell modulation. Moreover, PAS-induced plasticity seems to be heavily influenced by attention [35]. Lastly, it could be hypothesized that since CBI measurements were conducted at rest, the extent of the effects produced by the cPAS25 protocol was not enough to affect resting-state pathways, producing no significant effects on CBI at rest. These findings emphasize the importance of considering the timing and order of neuromodulatory interventions relative to behavioural tasks, and suggest that cerebellar plasticity may be transient, context-sensitive, and task-dependent.

Furthermore, the dual anatomical pathways through which peripheral input reaches the cerebellum, via mossy and climbing fibers, may underlie the complex effects of cPAS. The inferior olivary pathway, which gives rise to climbing fibers, is particularly responsive to unexpected or externally triggered stimuli [37], making it well-suited to convey instructive signals for associative learning. When paired with cerebellar TMS, which is believed to activate Purkinje cells directly [38], the combined stimulation may reproduce the sensorimotor contingencies necessary to induce LTD-like plasticity. Additionally, the involvement of both the granular layer and the deep cerebellar nuclei, which receive processed inputs from mossy fibers, supports a model of distributed plasticity extending beyond the cerebellar cortex (D’Angelo et al., 2018).

From a translational perspective, our findings provide a critical bridge between physiological plasticity and functional motor outcomes. The cPAS25 protocol not only modulated CBI, i.e. our physiological marker of cerebellar–cortical interaction but also improved motor skill acquisition. The absence of changes in MEP amplitude suggests that the observed effects were specific to cerebellar excitability, rather than global alterations in corticospinal excitability. This physiological specificity underscores the promise of cerebellar-targeted neuromodulation as a precise tool for motor rehabilitation.

While our study provides compelling evidence for the efficacy of cPAS25, several important questions remain unsolved. First, we assessed CBI modulation at a single post-stimulation time point, leaving the temporal dynamics and durability of these effects unknown. Future work should characterize the longevity and potential decay of cPAS-induced plasticity. Second, although our within-subject crossover design enhances internal validity, the sample was limited to healthy young adults. Testing cPAS25 in individuals with cerebellar dysfunction (e.g., ataxia, dystonia) will be critical to assess its translational potential. Third, the dose–response characteristics of cPAS remain unexplored. In other words, whether repeated sessions produce cumulative or more enduring effects is an open question. In both experiments, factors that can influence the CBI effect, such as attentional state and state dependence, were not analysed and therefore this represents a limitation. Future studies should take these factors into consideration.

Although MEP amplitude did not change in the present study (suggesting that M1 excitability remained stable), we did not re-evaluate RMT post-cPAS. This represents a methodological limitation.

Furthermore, the present study evaluated CBI using 60% of the MSO as the intensity of the cerebellar CS. This choice was made to replicate the exact same conditions used in the previous study (Ginatempo et al., 2024). Future studies should try assessing cPAS effects on CBI adjusting the CS intensity to the threshold of each participant. Finally, incorporating neuroimaging or electrophysiological methods (e.g., fMRI, EEG) could provide mechanistic insights into how cerebellar modulation influences broader motor networks during learning.

### Conclusion and Summary

In summary, our study demonstrates that cerebellar PAS delivered at a physiologically relevant interval (cPAS25) induces both neurophysiological plasticity and behavioural enhancement in young healthy adults. These findings underscore the cerebellum’s role in gating motor learning through timing-dependent mechanisms and support the use of targeted neuromodulation to engage these circuits. The clear dissociation from cPAS10 further validates the temporal specificity of the protocol. cPAS25 represents a promising tool for experimental and therapeutic modulation of cerebellar–cortical pathways, with implications for neurorehabilitation, learning enhancement, and the broader understanding of human motor control.

## Data Availability

The data that support the findings of this study are made available from the corresponding author, upon reasonable request.
